# Solitary Osteochondroma Arising from Cervical Spina Bifida Occulta

**DOI:** 10.1155/2013/509745

**Published:** 2013-12-08

**Authors:** Ali Ender Ofluoglu, Anas Abdallah, Akin Gokcedag

**Affiliations:** Department of Neurosurgery, Bakirkoy Research and Training Hospital for Neurology Neurosurgery, and Psychiatry, 34147 Istanbul, Turkey

## Abstract

Solitary osteochondromas are common benign long bone tumors originating from cartilage. They may produce a wide variety of symptoms and complications depending on their spinal location. These may include compressive myelopathy, nerve root compression, pathologic fracture and malignant degeneration, or in some cases only pain. Solitary cervical spine osteochondromas have been reported mostly in the neural arch or vertebral body. This report describes a patient presenting with neck pain, with a benign osteochondroma arising in the right bifid C5 lamina.

## 1. Introduction

Solitary osteochondromas are common lesions and account for 30–40% of all benign bone tumors [1]. Osteochondromas typically affect the long bones either in a solitary form or in multiple form known as multiple exostosis or osteochondromatosis [2, 3]. Spinal osteochondromas are rare lesions that make up less than 4% of spinal neoplasms [4–7]. Spinal osteochondroma rarely causes spinal cord compression and neurological symptoms such as radiculopathy and myelopathy [2, 3, 5]. The age of onset for spinal osteochondromas is 20–30 years with predominance in males [5, 6]. Solitary spinal osteochondromas may produce a wide variety of symptoms and complications depending on their location and relationship to associated structures. These may include compressive myelopathy, nerve root compression, pathologic fracture and malignant degeneration, or in some cases only pain. Solitary cervical spine osteochondromas have been reported mostly in the neural arch or vertebral body. This case report describes a patient presenting with neck pain, with a benign osteochondroma arising in the right bifid C5 lamina.

## 2. Case Report

A 28-year-old male patient presented to our clinic with neck pain. He had neck and right upper extremity pain for two years. Pain was especially on the right shoulder and was extending to the right wrist and hand. His past medical history was unremarkable with no trauma reference. He experienced no benefit from medical treatment. His complaints increased during the last 1.5 months. We admitted the patient to our clinic for further examination and treatment.

At physical examination, cervical neck movements were limited and painful in all four directions and there was local tenderness on the right side of C5 and C6 dermatomes. Muscular strengths were normal. There was no sensorial deficit. Deep tendon reflexes were all normal. Hoffman reflex and clonus were bilaterally negative.

**Figure 1:**
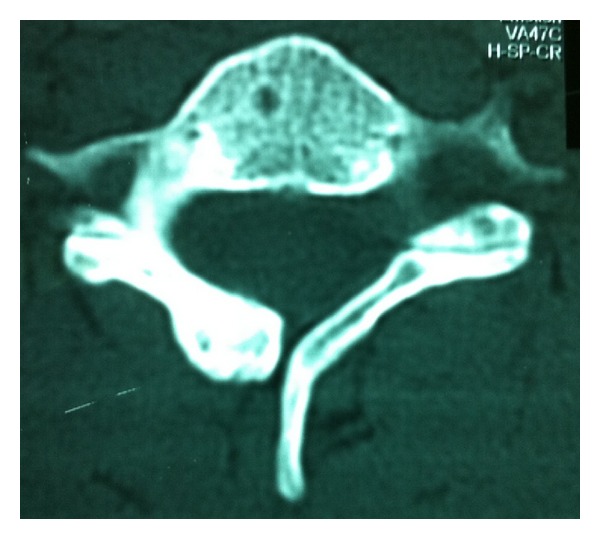
CT image of the lesion. Lamina bifida of C5 and the expansile sclerotic lesion on it are seen.

**Figure 2:**
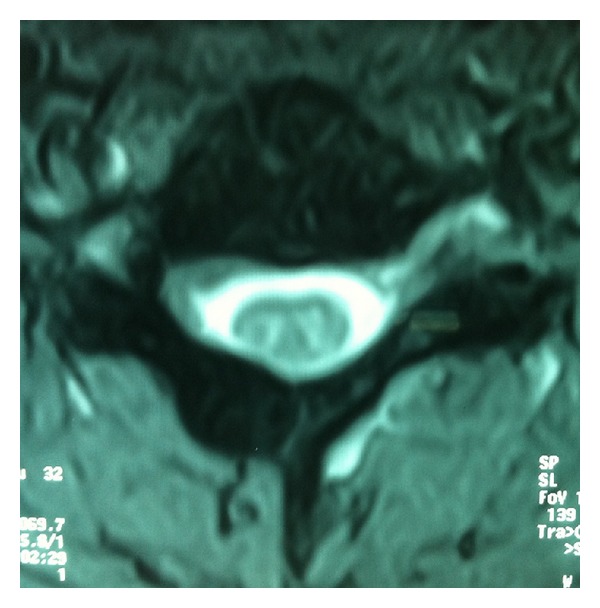
Mild expansion on the lamina of C5 vertebra and hypointense sclerotic lesion seen on T2 weighted MRI image.

The patient was referred to the radiology department for further evaluation of the spinal region. Cervical radiographs, computed tomography, and magnetic resonance imaging were performed. No significant lesion was detected in anteroposterior and lateral radiographs. In cervical CT (computerized tomography), there were coarsening of bifid lamina of C5 vertebra and a hyperintense expansile lesion on the lamina (Figure 1). In cervical spinal MRI (magnetic resonance imaging), there was mild expansion on lamina of C5 vertebra and there was a lesion that was hypointense on both T1 and T2 weighted images and compatible with a sclerotic, osteoblastic lesion like osteoma (Figure 2).

**Figure 3:**
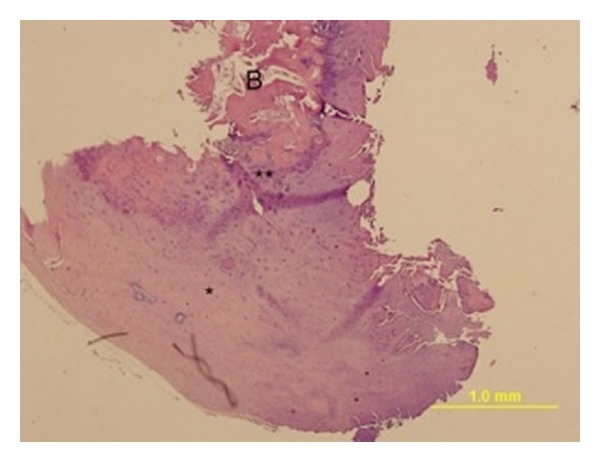
Photomicrograph of the resected lesion shows the bluish cartilage cap (*) demarcated from the underlying trabecular bone (B) by the secondary ossification line (**). Hematoxylin and eosin sections.

The patient was operated with the primary diagnosis of an expansile sclerotic, osteoblastic lesion on the bifid lamina of C5 vertebra. During the operation, a vertical incision was performed between the levels of spinous processes of C3 and C7 vertebrae. Bifid lamina of C5 vertebra was seen and the expansile lesion on the lamina was removed microscopically following a right-sided hemilaminectomy. The tumoral lesion was reported as osteochondroma in the histopathological examination (Figure 3). Skeletal scintigraphy demonstrated mild radiotracer uptake only on the right C5 vertebral lamina.

The patient had no neurological deficits after surgical treatment. His pain on the right upper extremity disappeared just after the surgery. The patient was then discharged and recommended for clinical control.

## 3. Discussion

Osteochondromas or osteogenic exostoses can be both solitary and multiple as in the case of hereditary familial exostosis. Osteochondromas represent 30–40% of all benign bone tumors [1]. Osteochondromas typically affect the long bones either in a solitary form or in a multiple form known as multiple exostosis or osteochondromatosis [2, 3]. Spinal osteochondroma is an uncommon entity; its frequency varies between 1 and 4% for the solitary form and 7 and 9% for the multiple form [8]. A spinal osteochondroma (exostosis) is a protrusion of a well-circumscribed, protruding mass of the neural arch [9]. This mass has a bony stalk that is pedunculated or sessile, and it is covered by a cartilaginous cap [9]. Cartilaginous cap increases in size with the active growing during normal bone growth, both in childhood and adolescence [10, 11].

Spinal osteochondromas are generally seen at the cervical and thoracic regions [1, 3, 8]. All levels of vertebral column can be involved, but the site most affected by isolated osteochondromas is C1 and for the multiple forms C2 [12, 13]. Spinal osteochondromas usually arise from the posterior elements that are the secondary ossification centers, and most commonly near the tips of the spinous processes [10]. Spinal cord compression due to a solitary or multiple exostosis is rare [11]. They are mostly asymptomatic; for that reason the diagnosis is generally delayed. The symptoms occur when the tumor compresses the spinal cord, nerve roots, or surrounding structures [3]. Spinal cord compression is more frequent in the familial forms than in the solitary forms [2, 13]. The most common symptoms are radiating pain, motor deficits, sensory disturbances, and urinary incontinence [3]. The clinical signs and symptoms appear in second and third decades of life, with an average age of 20 at the time of diagnosis [13, 14]. In our case, the solitary osteochondroma arose from the right posterior element of C5 vertebra and from the bifid lamina, and it caused compressive radicular symptoms in the right upper extremity. Our patient had complaints due to osteochondroma approximately since 25 years of age, and he was diagnosed at 28 years of age.

The detection of spinal osteochondromas is difficult on plain radiographs because of the complex image formed by the spinal bony elements [10, 13]. CT is the choice for diagnosis due to its convenience in revealing the cartilaginous and osseous structures of the lesion [3, 10, 13]. MRI is more useful than CT to define the relationship between tumor and the neighboring structures like dural sheath and it also shows the spinal cord compression [10, 13]. The main complication of osteochondroma is malignant transformation, not the local spinal cord compression. The risk for this is 1–5% for solitary forms and 10–25% for multiple forms [15]. For that reason, a detailed clinical and radiological investigation must be done for all patients with osteochondromas. In our case, we diagnosed the osteochondroma of the patient with radiography, and we performed CT and MRI for definitive diagnosis before surgery and also for more detailed evaluation of the tumor, its extensions and its relations with the surrounding structures.

In case progressive neurological symptoms develop, the treatment of choice for spinal osteochondroma is surgical removal of the tumor [3]. The management of this case was aimed at relieving neurological compromise. Total removal of the tumor is recommended where possible, because incomplete excision of the cartilaginous cap can lead to tumor recurrence [1, 15]. In most cases, the surgical outcome is good and laminectomy is the most common treatment method [4]. However, postlaminectomy kyphosis can be seen. Therefore, laminectomy should be minimized to prevent this, especially in young patients like our patient [14]. In our case, we surgically removed the lesion totally with right hemilaminectomy and the complaints of the patient due to the tumor gradually decreased and disappeared just after the surgery. We did not observe any early follow-up complications or sequelae after surgery.

## 4. Conclusion

Spinal osteochondromas are rare entities and clinical manifestations due to spinal cord compression by the tumor are rarely seen. Total removal of the tumor is the choice of treatment and it is a must for avoiding the recurrence of the tumor. Partial hemilaminectomy must be chosen especially in younger patients to prevent kyphosis due to laminectomy.
